# Impact of ^18F^FDG-PET/CT and Laparoscopy in Staging of Locally Advanced Gastric Cancer: A Cost Analysis in the Prospective Multicenter PLASTIC-Study

**DOI:** 10.1245/s10434-024-15103-4

**Published:** 2024-03-25

**Authors:** Cas de Jongh, Miriam P. van der Meulen, Emma C. Gertsen, Hylke J. F. Brenkman, Johanna W. van Sandick, Mark I. van Berge Henegouwen, Suzanne S. Gisbertz, Misha D. P. Luyer, Grard A. P. Nieuwenhuijzen, Jan J. B. van Lanschot, Sjoerd M. Lagarde, Bas P. L. Wijnhoven, Wobbe O. de Steur, Henk H. Hartgrink, Jan H. M. B. Stoot, Karel W. E. Hulsewe, Ernst Jan Spillenaar Bilgen, Marc J. van Det, Ewout A. Kouwenhoven, Freek Daams, Donald L. van der Peet, Nicole C. T. van Grieken, Joos Heisterkamp, Boudewijn van Etten, Jan-Willem van den Berg, Jean-Pierre Pierie, Hasan H. Eker, Annemieke Y. Thijssen, Eric J. T. Belt, Peter van Duijvendijk, Eelco Wassenaar, Kevin P. Wevers, Lieke Hol, Frank J. Wessels, Nadia Haj Mohammad, Geert W. J. Frederix, Richard van Hillegersberg, Peter D. Siersema, Erik Vegt, Jelle P. Ruurda, the PLASTIC Study Group

**Affiliations:** 1 Department of Surgery, Medical Oncology and Radiology, University Medical Center (UMC) Utrecht, Utrecht, The Netherlands; 2 Julius Center for Health Sciences and Primary Care, UMC Utrecht, Utrecht, The Netherlands; 3 Surgery and Nuclear Medicine Department, The Netherlands Cancer Institute-Antoni van Leeuwenhoek, Amsterdam, The Netherlands; 4 Surgery Department, Amsterdam UMC, Location University of Amsterdam, Amsterdam, The Netherlands; 5 Surgery and Pathology Department, Cancer Center Amsterdam, Amsterdam, The Netherlands; 6 Surgery Department, Catharina Hospital Eindhoven, Eindhoven, The Netherlands; 7 Surgery and Nuclear Medicine Department, Erasmus Medical Center UMC Rotterdam, Rotterdam, The Netherlands; 8 Surgery Department, Leiden UMC, Leiden, The Netherlands; 9 Surgery Department, Zuyderland MC, Sittard-Geleen, The Netherlands; 10 Surgery Department, Rijnstate Hospital, Arnhem, the Netherlands; 11 Surgery Department, ZGT Hospital, Almelo, The Netherlands; 12 Surgery and Pathology Department, Location Vrije University, Amsterdam UMC, Amsterdam, The Netherlands; 13 Surgery Department, Elisabeth Twee-Steden Hospital, Tilburg, The Netherlands; 14 Surgery Department, UMC Groningen, Groningen, The Netherlands; 15 Surgery Department, Medical Center Leeuwarden, Leeuwarden, The Netherlands; 16 Gastroenterology Department, Albert Schweitzer Hospital, Dordrecht, The Netherlands; 17 Surgery Department, Gelre Hospitals, Apeldoorn, The Netherlands; 18 Surgery Department, Isala Hospital, Zwolle, The Netherlands; 19 Gastroenterology Department, Maasstad Hospital, Rotterdam, The Netherlands; 20 Gastroenterology and Hepatology Department, Erasmus MC – University Medical Center, Rotterdam, Rotterdam, The Netherlands

**Keywords:** Gastric cancer, Costs, Staging, Positron emission tomography, Laparoscopy, Gastrectomy

## Abstract

**Background:**

Unnecessary D2-gastrectomy and associated costs can be prevented after detecting non-curable gastric cancer, but impact of staging on treatment costs is unclear. This study determined the cost impact of ^18^F-fluorodeoxyglucose positron emission tomography/computed tomography (^18F^FDG-PET/CT) and staging laparoscopy (SL) in gastric cancer staging.

**Materials and Methods:**

In this cost analysis, four staging strategies were modeled in a decision tree: (1) ^18F^FDG-PET/CT first, then SL, (2) SL only, (3) ^18F^FDG-PET/CT only, and (4) neither SL nor ^18F^FDG-PET/CT. Costs were assessed on the basis of the prospective PLASTIC-study, which evaluated adding ^18F^FDG-PET/CT and SL to staging advanced gastric cancer (cT3–4 and/or cN+) in 18 Dutch hospitals. The Dutch Healthcare Authority provided ^18F^FDG-PET/CT unit costs. SL unit costs were calculated bottom-up. Gastrectomy-associated costs were collected with hospital claim data until 30 days postoperatively. Uncertainty was assessed in a probabilistic sensitivity analysis (1000 iterations).

**Results:**

^18F^FDG-PET/CT costs were €1104 including biopsy/cytology. Bottom-up calculations totaled €1537 per SL. D2-gastrectomy costs were €19,308. Total costs per patient were €18,137 for strategy 1, €17,079 for strategy 2, and €19,805 for strategy 3. If all patients undergo gastrectomy, total costs were €18,959 per patient (strategy 4). Performing SL only reduced costs by €1880 per patient. Adding ^18F^FDG-PET/CT to SL increased costs by €1058 per patient; IQR €870–1253 in the sensitivity analysis.

**Conclusions:**

For advanced gastric cancer, performing SL resulted in substantial cost savings by reducing unnecessary gastrectomies. In contrast, routine ^18F^FDG-PET/CT increased costs without substantially reducing unnecessary gastrectomies, and is not recommended due to limited impact with major costs.

*Trial registration*: NCT03208621. This trial was registered prospectively on 30-06-2017.

**Supplementary Information:**

The online version contains supplementary material available at 10.1245/s10434-024-15103-4.

Gastric cancer is the third cause of cancer mortality worldwide with > 760,000 deaths in 2020.^1^ Treatment strategies depend on extent of disease at primary staging. Curative multimodality treatment consists of D2-gastrectomy combined with perioperative chemotherapy in most Western countries, resulting in 36–45% 5-year survival.^2–6^ However, this treatment is associated with considerable morbidity and substantial costs.^3–5,7–9^ If non-curable disease is detected, patients have no oncological benefit from surgical resection, and unnecessary D2-gastrectomy with its associated costs can be prevented. Hence, accurate staging to detect distant metastases and/or irresectable disease (cM1/cT4b) is essential for optimal patient selection for curative or palliative gastric cancer treatment.

Computed tomography (CT) of thorax and abdomen is routinely performed, but has limited accuracy for detecting cM1−/cT4b− stage, especially regarding peritoneal metastases.^10,11^ To increase diagnostic accuracy, ^18^F-fluorodeoxyglucose positron emission tomography with CT (FDG-PET/CT) and staging laparoscopy (SL) were incorporated in the Dutch national guidelines in 2016 for patients with locally advanced (cT3–4 and/or cN+) gastric cancer.^2^ Furthermore, NCCN guidelines and NICE guidelines also recommend performing ^18F^FDG-PET/CT if metastatic disease is absent on CT, similar to the Dutch national guidelines.^12,13^ In the Dutch multicenter PLASTIC-study, we prospectively evaluated implementation of these modalities and concluded that the added value of routine FDG-PET/CT is limited in detecting distant metastases (3%), whereas SL added considerably by detecting M1-/T4b- disease (19%), thereby substantially reducing futile surgery.^14^

Only one previous (single-center) study performed an economic evaluation of FDG-PET/CT and SL, suggesting a cost benefit for both modalities independently by reducing futile surgery and associated costs.^15^ This is in contrast to the PLASTIC-study findings, which strongly support SL but not routine use of FDG-PET/CT. Hence, the cost impact of these modalities remain unclear. The current study determined the cost impact of FDG-PET/CT and SL in gastric cancer staging by reducing unnecessary gastrectomies in the PLASTIC-study.^16^

## Materials and Methods

For this cost analysis, a decision tree was developed on the basis of the PLASTIC-study to determine the cost impact of performing FDG-PET/CT and SL in primary staging of advanced gastric cancer for all PLASTIC-patients by reducing futile gastrectomies after detecting non-curable disease. The analysis was performed from a healthcare perspective on the basis of hospital resource use. The time horizon dated from inclusion until 30-day postoperative follow-up, and until 90 days as alternative scenario. Because of this short time horizon, results are not discounted.

### PLASTIC-Study

The PLASTIC-study (2017–2020) was a prospective multicenter observational cohort study in 18 Dutch hospitals and assessed the added value of FDG-PET/CT and SL in staging locally advanced gastric cancer (cT3–4 and/or cN+) after initial staging with CT.^14,16^ The PLASTIC-study protocol and findings were published previously, detailing the inclusion criteria, primary staging, diagnostic criteria for irresectable or metastatic disease, and treatment procedures according to Dutch national guidelines based on TNM-7, and when available TNM-8.^2,14,16–18^ The timing of D2-gastrectomy with curative intent was within 6 weeks after completing neoadjuvant chemotherapy or after histopathological diagnosis in case of upfront surgical resection. Protocol mandated that FDG-PET/CT was performed first, followed by SL if no metastases were detected on FDG-PET/CT. Institutional review board approval was obtained at all centers and written informed consent was obtained for all patients.

### Structure of the Decision Tree

**Fig. 1:**
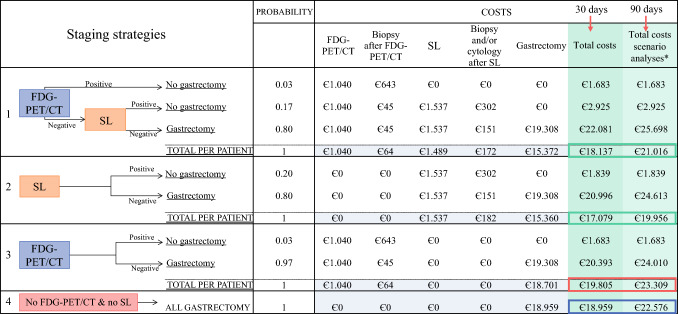
Decision tree to assess the cost impact of staging laparoscopy (SL) and FDG-PET/CT by modelling four staging strategies: (1) FDG-PET/CT first, then SL, (2) SL only, (3) FDG-PET/CT only, and (4) no SL nor FDG-PET/CT

To assess the cost impact of FDG-PET/CT and SL, four staging strategies were modeled in a decision tree to calculate total costs per strategy: (1) FDG-PET/CT first, then SL, (2) SL only, (3) FDG-PET/CT only, and (4) neither SL nor FDG-PET/CT (Fig. 1). Each branch of the decision tree depicts a negative outcome (no metastatic/irresectable disease) or positive outcome (detected metastases and/or irresectable disease) of FDG-PET/CT or SL after biopsy/cytology. The probability of a positive/negative outcome after FDG-PET/CT and SL was calculated using the observed frequency in the PLASTIC-population. Per staging strategy, patients with negative outcomes were distributed in the branch to undergo D2-gastrectomy (curative intent), whereas no gastrectomy was performed after positive outcomes (palliative treatment). Hence, the prospective data from the PLASTIC-study were used to predict the cost impact for each staging strategy in a theoretical model. If PLASTIC-patients with a positive SL were still treated with curative intent and underwent D2-gastrectomy (patients with positive cytology with no or limited macroscopic peritoneal dissemination), these patients were stratified into the decision tree according to their treatment (curative or palliative).

### Cost Parameters

Reimbursement prices issued by the Dutch Healthcare Authority (DHA) or national reference prices from the Dutch guideline on costing research in healthcare were used.^19–21^ As no standardized unit price for SL was available in the Netherlands and literature, SL costs were calculated bottom-up using hospital resource use (claim) data. Gastrectomy-associated costs were also calculated bottom-up. Claim data were collected from all PLASTIC-centers, and included surgery, hospital and intensive care unit admissions, diagnosis (imaging) and treatment of complications, surgical/endoscopic/radiological re-interventions, biochemical and laboratory tests, medication, and consultations and visits to outpatient clinics and emergency departments. Resource use from outside the hospital was not collected.

### Costs of FDG-PET/CT, Cytology, and Biopsy

For FDG-PET/CT, biopsy, and cytology, the DHA unit prices were used.^19,20^ The per patient averaged unit costs of biopsy/cytology after FDG-PET/CT included the diagnostic intervention, pathological assessment, and hospital admission for post-interventional observation.

### Costs of Staging Laparoscopy

SL costs were calculated bottom-up and included costs of surgery, SL-associated costs based on claim data from the PLASTIC-population, and costs of biopsy or peritoneal lavage including cytology.

Surgery costs were estimated on the basis of operating room costs using mean duration of SL in the PLASTIC-cohort and costs of surgical instruments, reusable materials, and laparoscopic equipment per SL based on purchasing prices, write-offs, and maintenance costs.^22^

Costs associated with SL included hospital admission for post-SL observation added with SL-related complications, which were prospectively recorded in the PLASTIC-study. SL-related complications were expressed in average costs per patient by assessing all hospital resource use during hospitalization following SL.

### Costs of D2-Gastrectomy

D2-gastrectomy costs consisted of previously reported costs of surgery added to postoperative gastrectomy-associated costs.

Surgery costs were based on a previous bottom-up calculation for D2-gastrectomy in the Dutch prospective LOGICA-trial, which included costs of the operating room, personnel salary, surgical instruments, laparoscopic equipment, disposable materials, and epidural placement in ten hospitals.^8^

Gastrectomy-associated costs were calculated using claim data from the PLASTIC-cohort. Costs of surgical re-interventions due to gastrectomy-related complications were calculated by multiplying the mean duration of re-operations (in min) in the PLASTIC-cohort with a previously reported €22 operating room min price, in addition to (sterilization) costs of surgical instruments.^22^

### Outcomes

The main outcome was the net cost impact per staging modality, calculated by comparing costs for performing FDG-PET/CT or SL versus cost savings by preventing unnecessary gastrectomies and associated costs. To compare costs per staging strategy, the total costs for each decision tree branch were multiplied by the proportion of patients per branch.

### Statistical Analysis

A probabilistic sensitivity analysis with 1000 iterations was performed to estimate uncertainty in net cost impact by comparing staging strategy 2 (SL only) versus strategy 1 (first FDG-PET/CT, then SL). This was illustrated in a violin plot where the width of the plot represents the number of iterations at that cost level, also including a boxplot. Statistical analyses were performed using R version 4.0.3 (R Foundation for Statistical Computing, Vienna, Austria) with RStudio (Boston, MA, USA).

## Results

### Study Population

**Fig. 2:**
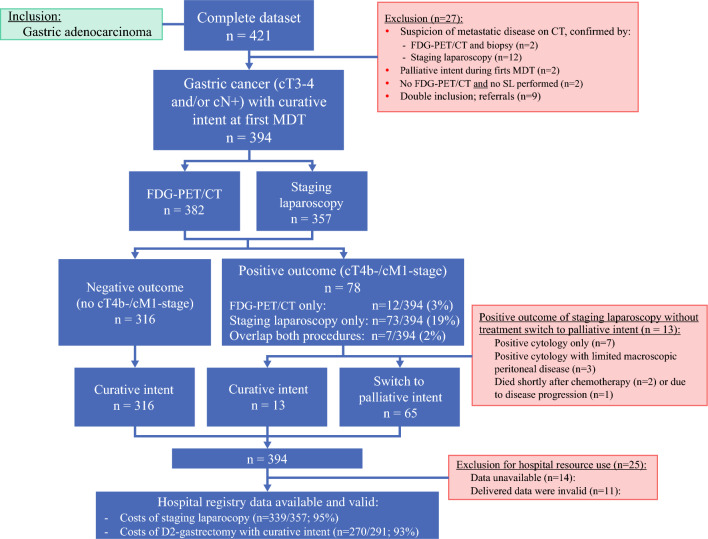
Flow chart of the study population and hospital claim data on hospital resource use

All 394 PLASTIC-patients from 18 hospitals included between August 2017 and February 2020 were analyzed in this cost analysis (Fig. 2). Their characteristics were previously reported.^14^ FDG-PET/CT was performed in 382 patients (97%) and 357 patients (91%) underwent SL, resulting in positive outcomes (cT4b−/cM1− stage) in 78/394 patients (20%). FDG-PET/CT was positive for distant metastases in 12/394 patients (3%) and SL was positive for peritoneal or irresectable disease in 73/394 patients (19%), with 2% overlap of cT4b−/cM1− disease detected by both procedures (*n* = 7/394; 2%). Details on the diagnostic performance of FDG-PET/CT and SL were previously reported.^14^

In 65 of these 78 patients (65/394; 16%), treatment was changed from curative to palliative intent (i.e., no D2-gastrectomy), including all 12 FDG-PET/CT-positive patients and 60/73 SL-positive patients. The remaining 13 SL-positive patients continued for curative treatment due to positive cytology with no (*n* = 7) or limited (*n* = 3) macroscopic peritoneal disease and underwent D2-gastrectomy, and three patients died before undergoing gastrectomy. In total, 329 patients (84%) were treated with curative intent, of whom 88% (*n* = 291/329) underwent D2-gastrectomy.

Hospital resource use data were unavailable for 14 patients (4%) from one center and delivered claim data were invalid for 11 patients (3%). The remaining claim data (Fig. 2) were used to calculate SL-related costs in 339/357 SL-patients (95%) and gastrectomy-associated costs in 270/291 gastrectomy patients (93%).

### Costs per FDG-PET/CT

Total costs per FDG-PET/CT were €1104 (Fig. 1), consisting of the DHA tariff per FDG-PET/CT (€1040) and mean costs per patient for additional procedures after FDG-PET/CT (€64). If cytology/biopsy (€151 DHA unit price) was performed (*n* = 22/382; 6%), the post-interventional hospital admission with a DHA-tariff of €476 per day was included. In nine patients (2%), magnetic resonance imaging (MRI; €380 DHA unit price) was performed after FDG-PET/CT to exclude/confirm distant metastases. The distribution of metastases and secondary findings (intra- and extra-abdominal) detected by FDG-PET/CT was previously reported.^14^

### Costs of Staging Laparoscopy

The bottom-up calculations estimated €1537 total costs per SL (Table 1), consisting of surgery costs (€975) for the operating room, disposable materials, surgical instruments and laparoscopic equipment, and SL-related costs (€562) for post-SL admission and SL-related complications, respectively. Performing peritoneal lavage including cytology (*n* = 264/357 SL patients; 74%) or biopsy (*n* = 115/357 SL patients; 32%) during SL to exclude/confirm distant metastases (€151 DHA tariff) added on average €172 per patient, totaling SL costs at €1709. All details from the SL bottom-up calculations are summarized in Supplementary Results.

**Table 1:** Bottom-up calculations of staging laparoscopy

Bottom-up calculations of staging laparoscopy estimated per patient (in euros)	*n* = 357 (100%)
*Surgery costs of staging laparoscopy*	
Minute price of operating room (€22) per mean duration of SL (33 min^a^) (including maintenance, personnel salary, and overhead)	€726
Disposable materials, *per SL*	€150
Trocars (1 × 10 mm, 2 × 5 mm)	€93
Rinse and suction system	€57
Reusable surgical instruments, *averaged per SL costs*^*b*^	€82
Sterilization costs	€80
Full surgical instrument set	€2
Laparoscopic equipment, *averaged per SL costs*^*b*^	€17
Laparoscope (endo eye 30°)	€4
Camera head	€2
Light source	€2
Video processor	€2
Insufflator	€1
Trolley	€1
Two monitors	€4
Suspension system	€1
**Subtotal costs**	**€975**
*Staging-laparoscopy-related costs*	
Hospital admission post-SL, *per day*	€476
SL-related complications^c^, *mean costs per patient based on hospital resource use*^d^	€86
**Subtotal costs**	**€562**
**Total costs per staging laparoscopy (without cytology/biopsy)**	**€1537**
*Additional averaged costs per patient for biopsy (n = 115/357; 32%) and/or cytology (n = 264/357; 74%) during SL*	+ €172

All amounts were rounded and calculated in euros (€)

SL: Staging laparoscopy, DHA: Dutch Healthcare Authority

^a^The surgical duration of staging laparoscopy was missing for 111 of the 357 patients (31%)

^b^To calculate averaged costs per SL, the total of the purchasing prices added to standard hospital maintenance costs were divided by write-offs. Write-offs were calculated using a 10-year life span (standard hospital policy) and the estimated number of times SL could theoretically be performed per business day in the operating room

^c^Three patients (< 1%) developed an SL-related complication, all requiring reoperation and prolonged hospital admission and diagnostic and therapeutic procedures, of which the costs totaled €28,551, thus averaging €86 per patient

^d^Hospital resource use data were missing for 18 patients (5%)

### Costs per D2-Gastrectomy

Total costs per D2-gastrectomy (€19,308; Table 2) consisted of surgery costs (€7354) and gastrectomy-related costs until 30 days after surgery (€11,605 hospital costs and €349 surgical re-interventions). In the additional scenario analysis using a follow-up of 90 days postoperatively (Table 2), total costs were €22,925 per D2-gastrectomy (€15,222 hospital costs and €349 surgical re-interventions). A detailed overview of surgical costs and hospital resource use following gastrectomy is provided in Supplementary Results.

**Table 2:** Costs of D2-gastrectomy and D2-gastrectomy-associated costs based on all hospital resource use within 30 days postoperatively (and 90 days as scenario analysis)

Bottom-up calculations of D2-gastrectomy per patient (in euros)	*n* = 291 patients (100%)	
Surgery costs of D2-gastrectomy	Unit price in a previous multicenter trial^a^	Frequencies in the PLASTIC Study^b.^
Laparoscopic total D2-gastrectomy	€8124	34%
Laparoscopic distal D2-gastrectomy	€7353	37%
Open total D2-gastrectomy	€6584	15%
Open distal D2-gastrectomy	€5893	15%
**Subtotal costs**	**€7354 per D2-gastrectomy (PLASTIC study)**	

D2-gastrectomy related costs (based on hospital resource use)	30-day postoperative follow-up	90-day postoperative follow-up
Hospital admission, *per day*	€4726	€6201
Intensive care unit admission,* per day*	€3739	€4641
Imaging	€539	€818
Other diagnostics or interventions	€1982	€2325
Visits to outpatient clinics or paramedics	€602	€1041
Other costs	€17	€196
**Subtotal costs**	**€11,605**	**€ 15,222**
D2-gastrectomy-related costs (based on clinical PLASTIC-data)		
Surgical re-interventions^c^	€349	€349^d^
Subtotal gastrectomy-related costs	€11,954	€15,571
**Total costs per D2-gastrectomy in the PLASTIC-cohort**	**€19,308**	**€22,925**

Percentages may not add up to 100% due to rounding

^a^Unit prices of D2-gastrectomy in a previous prospective multicenter include the use of operating room, disposable and reusable materials, surgical instruments, and laparoscopic equipment, as reported by Van der Veen et al.^8^

^b^The surgical approach (open or laparoscopic) was missing for 36 patients (12%)

^c^The costs of surgical re-interventions were calculated by multiplying the duration of surgery (this could be retrieved for 32% of reoperations) by the €22 minute price of the operating room as previously reported by Bolkenstein et al.^20^ As 14% of operated patients underwent surgical re-intervention (€2478), the average cost per patient totaled €349

^d^Surgical re-interventions were recorded until 30 days after surgery

### Cost Impact of FDG-PET/CT and SL (Decision Tree)

Costs of the four strategies are displayed in Fig. 1. The decision tree input parameters are listed in Table 3. The mean total costs per patient for each strategy were €18,137 for strategy 1 (first FDG-PET/CT, then SL), €17,079 for strategy 2 (SL only), €19,805 for strategy 3 (FDG-PET/CT only), and €18,959 for strategy 4 (no FDG-PET/CT and no SL), respectively.

**Table 3:** Input parameters for the decision tree

Transition probabilities	Value
Sensitivity FDG-PET/CT + biopsy	0.40
Sensitivity staging laparoscopy after FDG-PET/CT	0.85
Sensitivity staging laparoscopy	0.87
Transition FDG-PET/CT, palliative patients	0.03
Transition staging laparoscopy, palliative patients (after negative FDG-PET/CT)	0.18
Transition staging laparoscopy, palliative patients (no FDG-PET/CT)	0.20

Costs (*per unit*)	Amount per patient (in euros)
FDG-PET/CT	€1040
Biopsy/cytology because of findings on FDG-PET/CT	€643
Staging laparoscopy	€1451
Complication after staging laparoscopy	€9517 (€86 per patient)
Biopsy/cytology because of findings during staging laparoscopy	€151
D2-gastrectomy	€7354
Surgical re-intervention	€2478 (€349 per patient)
D2-gastrectomy-related costs (30 days post-surgery)	€11,605
D2-gastrectomy-related costs (90 days post-surgery)	€15,222

Other parameters	Value (in %)
% Biopsy/cytology after FDG-PET/CT—curative intent	7.03%
% Cytology after staging laparoscopy—curative intent	73.95%
% Biopsy after staging laparoscopy—curative intent	32.49%
% Complication after staging laparoscopy	0.90%
% Positive outcome of staging laparoscopy—palliative intent	82.19%
% Surgical re-intervention after D2-gastrectomy	14.09%

These input parameters were used in the decision tree (Table 1) to calculate the total costs for each of the four staging strategies.

After calculating the additional costs for performing FDG-PET/CT and/or SL, and subtracting this with the reduction in costs by preventing futile gastrectomies, the net cost impact was calculated per staging strategy (Table 4). Compared with strategy 4 (without FDG-PET/CT and SL) in which all patients would undergo D2-gastrectomy, performing SL only (strategy 2) was the most cost-beneficial strategy with net cost savings of €1880 per patient. Performing FDG-PET/CT first and then SL increased costs by €1058 per patient, but still resulted in net cost savings of €822 per patient. In contrast, strategy 3 (FDG-PET/CT only) showed a net cost increase of €847 per patient.

**Table 4:** Net cost impact of different staging strategies with FDG-PET/CT and/or staging laparoscopy

Net cost impact of the different staging strategies; all strategies are compared with strategy 4: neither FDG-PET/CT nor staging laparoscopy		Amounts per patient (in euros)^a^	
Staging strategy 1: first FDG-PET/CT, then staging laparoscopy			
Additional costs for performing staging modalities		+€2765	
Cost savings by preventing unnecessary D2-gastrectomy		−€3587	
Net cost impact strategy 1	Cost savings	−€822	Per patient
	Cost savings in the Netherlands, each year	−€452,100	(*n* = 550)^b^
Staging strategy 2: only staging laparoscopy			
Additional costs for performing staging modalities		+€1719	
Cost savings by preventing unnecessary D2-gastrectomy		−€3599	
Net cost impact strategy 2	Cost savings	−€1880	Per patient
	Cost savings in the Netherlands, each year	−€1,034,000	(*n* = 550)^b^
Staging strategy 3: only FDG-PET/CT			
Additional costs for performing staging modalities		+€1104	
Cost savings by preventing unnecessary D2-gastrectomy		-€257	
Net cost impact strategy 3	Cost increase	+€847	Per patient
	Cost increase in the Netherlands, each year	+€465,850	(*n* = 550)^b^

^18F^FDG-PET/CT: ^18F^Flurodeoxyglucose positron emission tomography/computed tomography

^a^These results are from the 30-day postoperative follow-up scenario. The 90-day follow-up scenario yielded similar cost differences per patient with a net cost reduction of €2620 for strategy 2 and €1560 for strategy 1, and a net cost increase of €733 for strategy 3

^b^The yearly cost savings in the Netherlands were based on an estimated 550 patients diagnosed with advanced gastric cancer (cT3–4 and/or cN+) without distant metastases at initial CT scans.^19^

Translated into national perspective in the Netherlands, with an incidence of approximately 550 patients with advanced gastric cancer without distant metastases at CT, this would result in annual cost savings of approximately €1,034,000 when performing only SL, and €452,100 when adding FDG-PET/CT to SL (Table 4), respectively.^23^ In contrast, performing only FDG-PET/CT would lead to a yearly cost increase of approximately €465,850.

In the scenario analysis using 90-day follow-up for gastrectomy-associated costs (Table 2), total cost differences per patient were greater than the 30-day scenario: the net cost savings were €2620 for strategy 2 and €1560 for strategy 1, and the net cost increase was €733 for strategy 3.

### Probabilistic Sensitivity Analysis

**Fig. 3:**
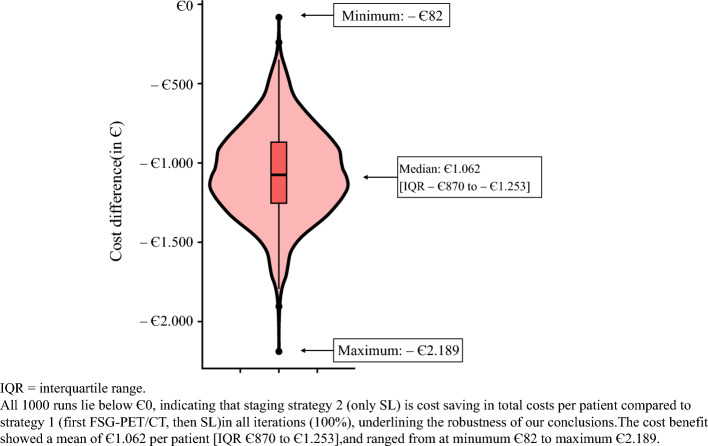
Violin plot displaying the probabilistic sensitivity analysis that compared the net cost savings of staging strategy 2 (only SL) versus strategy 1 (first FDG-PET/CT, then SL) in 1000 iterations

The probabilistic sensitivity analysis (Fig. 3) showed that all 1000 runs (100%) comparing staging strategy 2 (only SL) versus strategy 1 (first FDG-PET/CT, then SL) resulted in net cost savings. The median cost benefit for the strategy ‘SL only' was €1062 per patient (IQR €870–1253), ranging €82–2189.

## Discussion

The present multicenter study determined the cost impact of FDG-PET/CT and SL in primary staging of locally advanced gastric cancer. The results demonstrated that routinely performing SL resulted in substantial cost savings by preventing futile D2-gastrectomies and associated costs due to detecting peritoneal metastases or irresectable disease during SL. In contrast, routine use of FDG-PET/CT increased total costs, as performing FDG-PET/CT showed limited impact and resulted in only few prevented futile gastrectomies. To the best of our knowledge, this is the largest prospective and first multicenter economic evaluation of the cost impact of FDG-PET/CT and SL in primary staging of advanced gastric cancer.

The staging strategy of performing only SL resulted in the highest cost benefit by considerably reducing unnecessary D2-gastrectomies. Adding FDG-PET/CT prior to SL resulted in extensive additional costs (€1058 per patient) without substantial added benefit in reducing futile surgery because of its limited diagnostic performance in detecting distant metastases. This cost analysis gives a monetary value to the clinical results from the Dutch prospective multicenter PLASTIC-study, which concluded that FDG-PET/CT has limited added value with 3% detected distant metastases, while SL substantially improves detection of non-curable disease (19%) in staging advanced gastric cancer, resulting in treatment changes from curative to palliative intent.^14^ If first SL and then FDG-PET/CT would be performed depending on the SL outcome, the FDG-PET/CT accuracy to detect distant metastases would be lower (1%), resulting in even higher FDG-PET/CT costs. Currently, Dutch national guidelines and international NCCN/NICE guidelines advise routine use of both FDG-PET/CT and SL in staging of locally advanced gastric cancer (cT3–4 and/or cN+), whereas the clinical PLASTIC-results combined with this cost analysis demonstrate that FDG-PET/CT should not be routinely performed for this population.^2,12,13,17,18^

In our study, the chosen 30-day postoperative follow-up probably leads to a minor underestimation of D2-gastrectomy costs, because a small proportion of patients is hospitalized or readmitted for complications beyond 30 days after surgery.^24,25^ The alternative 90-day follow-up scenario analysis covers these additional costs, however, it may provide a minor overestimation of gastrectomy costs, as some patients utilize hospital resources not directly related to D2-gastrectomy (e.g., other hospital visits). The 30-/90-day scenarios therefore provide a range in gastrectomy-related costs (€19,308–22,925). Importantly, both scenarios yielded equivalent conclusions. Other uncertainty was assessed in the probabilistic sensitivity analysis, which demonstrated that all 1000 iterations (100%) confirmed that staging with only SL is significantly more cost-beneficial than routinely combining FDG-PET/CT and SL.

Only one previous single-center study reported on healthcare costs of FDG-PET/CT and SL in gastric cancer staging, and found that first FDG-PET/CT and then SL was the more cost-efficient strategy (USD $13,571 per patient) than vice versa, while both modalities substantially reduced futile surgery independently.^15^ Although our results regarding SL are in line with this previous study, our FDG-PET/CT-findings demonstrated a much lower metastatic detection rate and substantially increased costs accordingly.^26^ Performing only SL clearly proved to be the most cost-efficient strategy, saving €1880 per patient. This difference in FDG-PET/CT costs could be explained by different definitions in T-staging between TNM-6 versus TNM-7/TNM-8 classifications, resulting in higher cT-stages in their study.^15,17,18,27,28^ Higher cT-stages may improve the pretest likelihood of finding metastases and increase FDG avidity.^14,27,29,30^ Furthermore, differences in healthcare systems may play a role, such as their very high gastrectomy costs (USD $144,000) which increase financial impact of preventing a single gastrectomy. However, in our study participated 18 centers, likely representing clinical practice more reliably.^14,15,31,32^

As no reference prices were available for SL, we calculated unit costs per SL (€1537) for surgery and SL-related admission and complications bottom-up, which increased to €1709 when adding peritoneal lavage including cytology and biopsy during SL.^19,20^ To our knowledge, these total costs per SL for gastric cancer is the most detailed approximation currently available.

Since there is currently no international consensus on the optimal treatment for patients with positive cytology with no or limited macroscopic peritoneal disease, we stratified these patients (*n* = 10) in the decision tree according to their treatment intent (curative or palliative).^6,18,33–36^ Previous studies demonstrated a survival benefit for curative treatment for this unique patient subgroup, especially when positive cytology converts into negative cytology after neoadjuvant chemotherapy.^34^ Results from ongoing clinical trials may lead to updated treatment strategies for this small patient subgroup.^37,38^

Several limitations should be addressed. First, cost-savings of futile D2-gastrectomy for palliative patients were calculated on the basis of hospital costs from curatively treated patients, which might differ from practice: not every positive SL would result in preventing full costs of D2-gastrectomy, because intraoperative detection of peritoneal metastases can result in aborting surgical resection. This would shorten surgery and may reduce morbidity, lowering gastrectomy costs. However, overall, our gastrectomy costs are probably underestimated, because no out-of-hospital costs (e.g., nursing or rehabilitation homes, general practitioner) were collected, which would increase gastrectomy-associated costs and thereby impact on preventing gastrectomy.^8^ Moreover, these populations might differ, for instance, frailty of palliative patients may increase morbidity and thus costs.^9,24,39,40^ Importantly, the 30-/90-day scenarios and probabilistic sensitivity analysis all yielded equivalent conclusions. Second, healthcare resource use and unit costs can differ per country, and as a consequence the cost calculations may yield different amounts among different healthcare systems. Although current literature lacks data on the variations in costs between countries for performing staging modalities and D2-gastrectomy, such potential cost differences were deemed to be minor, especially in other Western countries. Hence, the final conclusions and recommendations of our study are probably translateable to other countries. Third, this analysis focused on evident benefit of preventing unnecessary gastrectomy by detecting non-curable disease, however, other aspects were not included. For instance, incidental findings on FDG-PET/CT were not included as their added value is uncertain in literature and cannot be adequately determined.^14,41–44^ Furthermore, costs of systemic (chemo)therapy could not be incorporated. Theoretically, curative chemotherapy would result in higher costs than palliative systemic therapy due to intensified regimens and higher toxicity with related hospitalizations.^45^ Hence, detecting non-curable disease would further increase the differences in cost impact between SL and FDG-PET/CT, supporting our current conclusions. Overall, the present detailed cost analysis combined with clinical results from the prospective PLASTIC-study in 18 hospitals justifies revision of current staging guidelines. We recommend to standardly perform SL for all patients with advanced gastric cancer, whereas routine use of FDG-PET/CT should be abandoned, and FDG-PET/CT should be restricted to specific populations with higher risk of (finding) distant metastases.

Future studies could focus on patient selection for FDG-PET/CT on the basis of tumor characteristics, such as cT4−/cN+ tumors, intestinal type, gastroesophageal junction tumors, larger size, non-mucinous tumors, differentiation grade, or other factors associated with distant dissemination or FDG avidity on FDG-PET/CT.^15,26,29,30,46–50^ A previous study identified patient subgroups that could benefit from additional FDG-PET/CT-staging.^51^ However, this model has limited predictive value and needs further optimization. Additional studies are required to determine whether such approaches for FDG-PET/CT may be cost-effective.

In conclusion, for patients with locally advanced gastric cancer, performing SL resulted in substantial cost savings by considerably reducing futile D2-gastrectomies and associated costs. In contrast, routinely performing FDG-PET/CT increased total costs since few unnecessary D2-gastrectomies were prevented by FDG-PET/CT, saving few costs, while being expensive to perform. Routine use of FDG-PET/CT as staging tool for patients with advanced gastric cancer is not recommended, given the limited impact and substantially increased costs. Future studies may focus on identifying patient subgroups to increase the diagnostic performance of FDG-PET/CT, which could result in cost-beneficial strategies for FDG-PET/CT.

### Supplementary Information

Below is the link to the electronic supplementary material.

Supplementary file1 (PDF 143 kb)

## Data Availability

The data generated and/or analyzed during the study are available from the corresponding author upon reasonable request.
