# Supplementary material for: Impact of 18FFDG-PET/CT and Laparoscopy in Staging of Locally Advanced Gastric Cancer: A Cost Analysis in the Prospective Multicenter PLASTIC-Study
Source: Ann Surg Oncol. 2024 Mar 25;31(6):4005–17. doi: 10.1245/s10434-024-15103-4 (PMC11076388; doi:10.1245/s10434-024-15103-4)
Supplement: Supplementary file 1 — Supplementary file1 (PDF 143 kb) [file 10434_2024_15103_MOESM1_ESM.pdf]

Impact of ^{18F}FDG-PET/CT and laparoscopy in staging of locally advanced gastric cancer – a cost analysis in the prospective multicenter PLASTIC-study.

AUTHORS: Cas de Jongh[1] MD, Miriam P. van der Meulen[1*] MD PhD, Emma C. Gertsen[1] MD PhD, Hylke J.F. Brenkman[1] MD PhD, Johanna W. van Sandick[2] MD PhD, Mark I. van Berge Henegouwen[3,4] MD PhD, Suzanne S. Gisbertz[3,4] MD PhD, Misha D.P. Luyer[5] MD PhD, Grard A.P. Nieuwenhuijzen[5] MD PhD, Jan J.B. van Lanschot[6] MD PhD, Sjoerd M. Lagarde[6] MD PhD, Bas P.L. Wijnhoven[6] MD PhD, Wobbe O. de Steur[7] MD PhD, Henk H. Hartgrink[7] MD PhD, Jan H.M.B. Stoot[8] MD PhD, Karel W.E. Hulsewe[8] MD PhD, Ernst Jan Spillenaar Bilgen[9] MD PhD, Marc J. van Det[10] MD PhD, Ewout A. Kouwenhoven[10] MD PhD, Freek Daams[4,11] MD PhD, Donald L. van der Peet[4,11] MD PhD, NCT van Grieken[4,11] MD PhD, Joos Heisterkamp[12] MD PhD, Boudewijn van Etten[13] MD PhD, Jan-Willem van den Berg[13] MD PhD, Jean-Pierre Pierie[14] MD PhD, Hasan H. Eker[14] MD PhD, Annemieke Y. Thijssen[15] MD PhD, Eric J.T. Belt[15] MD PhD, Peter van Duijvendijk[16] MD PhD, Eelco Wassenaar[16] MD PhD, Kevin P. Wevers[17] MD PhD, Lieke Hol[18] MD PhD, Frank J. Wessels[1] MD PhD, Nadia Haj Mohammad[1] MD PhD, Geert W.J. Frederix[1*] MD PhD, Richard van Hillegersberg[1] MD PhD, Peter D. Siersema[19] MD PhD, Erik Vegt[2,6] MD PhD, Jelle P. Ruurda[1] MD PhD, on behalf of the PLASTIC Study Group.

INSTITUTIONS AND AFFILIATIONS: [1]University Medical Center (UMC) Utrecht, Utrecht, The Netherlands. [1*]Julius Center for Health Sciences and Primary Care, UMC Utrecht, Utrecht, The Netherlands. [2]The Netherlands Cancer Institute – Antoni van Leeuwenhoek, Amsterdam, The Netherlands. [3]Amsterdam UMC, location University of Amsterdam, The Netherlands. [4]Cancer Center Amsterdam, Amsterdam, The Netherlands. [5]Catharina Hospital Eindhoven, Eindhoven, The Netherlands. [6]Erasmus Medical Center UMC Rotterdam, Rotterdam, The Netherlands. [7]Leiden UMC, Leiden, The Netherlands. [8]Zuyderland MC, Sittard-Geleen, The Netherlands. [9]Rijnstate Hospital, Arnhem, The Netherlands. [10]ZGT Hospital, Almelo, The Netherlands. [11]Amsterdam UMC, location Vrije University, The Netherlands. [12]Elisabeth Twee-Steden Hospital, Tilburg, The Netherlands. [13]UMC Groningen, Groningen, The Netherlands. [14]Medical Center Leeuwarden, Leeuwarden, The Netherlands. [15]Albert Schweitzer Hospital, Dordrecht, The Netherlands. [16]Gelre Hospitals, Apeldoorn, The Netherlands. [17]Isala Hospital, Zwolle, The Netherlands. [18]Maasstad Hospital, Rotterdam, The Netherlands. [19]Radboud UMC, Nijmegen, The Netherlands.

SUPPLEMENTARY MATERIAL

Title page supplementary material Page no. 1

Supplementary Results 1. Costs per staging laparoscopy. Page no. 2 – 3

Supplementary Results 2. Costs per D2-gastrectomy. Page no. 3

Supplementary Results 1. Costs per staging laparoscopy.

The bottom-up calculations were €1,537 for total costs per SL, consisting of €975 of surgery costs and €562 of SL related costs for post-SL admission and complications, respectively (Table 1). In addition to the €1,537 bottom-up SL costs, 116 out of 357 SL-patients (32%) underwent histological biopsy and 264 SL-patients (74%) underwent cytology during SL, both with a DHA-tariff of €151 to confirm/exclude distant metastases (€172 averaged per patient), totaling SL-costs at €1,709.

Surgery costs per staging laparoscopy

Total surgery costs noted €975 per SL by counting costs of the use of the operating room (€726), disposable materials (€150), reusable surgical instruments (€82) and laparoscopic equipment (€17) per SL, respectively (Table 1).

The use of the operating room (€726 per SL) detailed the mean surgical duration of SL in the PLASTIC-study (33 minutes; n=246) multiplied with a previously reported €22 minute price of the operating room, which includes costs for using the operating room, maintenance, salary of surgical and anesthesiological personnel, and overhead.[20] SL operating time was known for 248 patients (69%), of whom two patients (1%) were excluded calculating the mean surgical duration because the full surgical resection was included in their operating time.

Costs of disposables (€150 per patient) were estimated based on hospital purchasing prices of trocars (€93) and the rinse and suction system (€57).

Reusable surgical instruments were estimated at €82 per SL and consisted of sterilization costs (€80) added with purchasing prices of the (reusable) surgical instruments (averaged €2 per SL). The instrument costs totaled €11,725 by adding the purchasing prices of one full standard SL instrument set (€6,700) plus 7.5% maintenance costs per year assuming a 10-years life-span (€5,025) according to standard hospital policy. By dividing this by the number of business days per year that these instruments were used, which was assumed to be 120 of the yearly 240 operating room business days, and the estimation that 6 SL can be performed per business day, these costs resulted in (rounded) €2 per SL on average.

Mean costs for using laparoscopic equipment during SL (€17) included costs for the laparoscope, camera head, light source, video processor, insufflator, trolley and two monitors with suspension system. Total costs numbered €119,459 by including purchasing prices (€56,255) and 7.5–15% maintenance costs for a 10-years lifespan (€63,204) according to standard hospital policy. Dividing this by 120 business days and 6 SL per business day, this resulted in €17 on average per SL.

Staging laparoscopy related costs

The SL related costs counted €562 per patient (Table 1) by adding the €476 DHA-tariff per day hospital admission post-SL with €86 of mean costs per patient for SL related complications.[17,18]

Three PLASTIC-patients developed a complication following SL (<1%), all requiring surgical re-intervention as published previously.[12] These complication costs totaled a mean of €9,517 per SL-related complication due to the surgery duration (in minutes) multiplied by the €22 previously reported operating room minute price, the prolonged hospital admission and the diagnostic and therapeutic procedures, resulting in additional costs of on average €86 per patient undergoing SL.

Supplementary Results 2. Costs per D2-gastrectomy.

Total costs per D2-gastrectomy (€19,308; Table 2) consisted of surgery costs (€7,354) and gastrectomy-related costs until 30 days after surgery (€11,605 hospital costs and €349 surgical re-interventions); or €22,925 per D2-gastrectomy up to 90 postoperative days as additional scenario analysis (€15,222 hospital costs and €349 surgical re-interventions).

The unit prices of total and distal D2-gastrectomy via laparoscopic and open approach were calculated bottom-up in a previous prospective randomized controlled trial at €8,124 for total and €7,353 for distal laparoscopic D2-gastrectomy, and €6,584 for total and €5,893 for distal open D2-gastrectomy.[8] Translated into the proportion performed total (34%) and distal (37%) laparoscopic and total (15%) and distal (15%) open D2-gastrectomies in the PLASTIC-study, the weighted average D2-gastrectomy unit price in the PLASTIC-cohort balanced €7,354 in total.

The gastrectomy-related costs from all 30-day postoperative hospital resource totaled €11,605, consisting of hospital (€4,726) and intensive care unit (€3,739) admissions, imaging (€539) and other diagnostics (€1,982) for complications, visits to outpatient clinics or paramedics (€602) and other costs (€17). For the 90-day scenario analyses, these costs totaled €15,222 by adding hospital (€6,201) and ICU (€4,641) admissions, imaging (€818) and other diagnostics (€2,325), visits to outpatient clinics or paramedics (€1,041) and other costs (€196).

The costs per surgical re-intervention totaled €2,478 at a mean duration of 109 minutes.[20] The surgery duration could be retrieved for 32% of reoperations. This translated into an average cost of €349 per patient as re-operations occurred in 14% of operated PLASTIC-patients.
